# Modulation of TNF‐α, interleukin‐6, and interleukin‐10 by nebivolol–valsartan and nebivolol–lisinopril polytherapy in SHR rats

**DOI:** 10.1002/prp2.1189

**Published:** 2024-03-19

**Authors:** Lezama‐Martinez Diego, Flores‐Monroy Jazmin, Ramirez‐Hernandez Diana, Garrido‐Farina German‐Isauro, Fonseca‐Coronado Salvador, Hernandez‐Campos Maria‐Elena

**Affiliations:** ^1^ Laboratory of Pharmacology, FES Cuautitlan Universidad Nacional Autonoma de Mexico Cuautitlan Izcalli Mexico Mexico; ^2^ Sección de Estudios de Posgrado e Investigación, Escuela Superior de Medicina Instituto Politecnico Nacional Federal District Mexico Mexico

**Keywords:** hypertension, IL‐10, IL‐6, nebivolol, polytherapy, TNF‐α

## Abstract

Antihypertensive drug therapies have demonstrated their capacity to modulate the inflammatory processes associated with hypertension, leading to improvements in disease progression. Given the prevalent use of polytherapy in treating most hypertensive patients, comprehending the time‐dependent effects of combination treatments on inflammation becomes imperative. In this study, spontaneously hypertensive rats (SHR) were divided into seven groups (*n* = 6): (i) SHR + vehicle, (ii) SHR + nebivolol, (iii) SHR + valsartan, (iv) SHR + lisinopril, (v) SHR + nebivolol–valsartan, (vi) SHR + nebivolol–lisinopril, and (vii) WKY + vehicle. Blood pressure was measured using the tail‐cuff method. Temporal alterations in inflammatory cytokines TNF‐α, IL‐6, and IL‐10 were assessed in serum through ELISA and mRNA expression in aortic tissue via qPCR after 1, 2, and 4 weeks of treatment with nebivolol, lisinopril, valsartan, and their respective combinations. Histological alterations in the aorta were assessed. The findings indicated that combined treatments reduced systolic and diastolic blood pressure in SHR. The nebivolol and lisinopril combination demonstrated a significant decrease in IL‐6 serum and mRNA expression at both 1 week and 4 weeks into the treatment. Additionally, TNF‐α mRNA expression also showed a reduction with this combination at the same time points. Particularly, nebivolol–valsartan significantly decreased TNF‐α serum and mRNA expression after one and four weeks of treatment. Furthermore, an elevation in serum IL‐10 levels was observed with both combination treatments starting from the second week onwards. This study provides compelling evidence that concurrent administration of nebivolol with lisinopril or valsartan exerts time‐dependent effects, reducing proinflammatory cytokines TNF‐α and IL‐6 while modifying IL‐10 levels in an experimental hypertensive model.

## Graphical abstract

Effect of polytherapy with beta‐blockers and renin‐angiotensin system inhibitors on IL‐6, IL‐10 and TNF‐α levels.

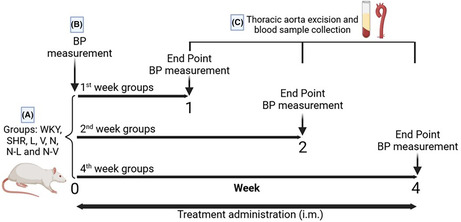

**Abbreviations**
ACEi: Angiotensin‐converting‐enzyme inhibitors
Ang II: Angiotensin II
ATR: Angiotensin receptor antagonist
DBP: Diastolic blood pressure
IL‐10: Interleukin‐10
IL‐6: Interleukin‐6
mRNA: Messenger ribonucleic acid
NF‐κB: Nuclear factor‐κB
RAS: Renin‐angiotensin system
SBP: Systolic blood pressure
SHR: Spontaneously hypertensive rat
TNF‐α: Tumor necrosis factor‐alpha
VMSC: Vascular smooth muscle cells
WKY: Wistar Kyoto rat

## 1 INTRODUCTION

Adequate pharmacological control of hypertension in patients is necessary to reduce the potential adverse effects associated with the development of the disease.^1^ In this regard, guidelines for the treatment of hypertension have established the need for the initial use of combination drug therapy to achieve adequate blood pressure control and reduce adverse effects.^2^

Hypertension is closely linked to the upregulation of various inflammatory mechanisms.^3^ A well‐established connection exists between essential hypertension and key inflammatory markers, including tumor necrosis factor‐alpha (TNF‐α), interleukin‐6 (IL‐6), and C‐reactive protein.^4^ Elevated proinflammatory cytokines, such as IL‐6 and TNF‐α, have been closely associated with organ damage in both human subjects and animal models of hypertension.^5^, ^6^, ^7^

IL‐6 assumes a pivotal role as a proinflammatory cytokine in this context. It exhibits an increase in plasma levels following angiotensin II (Ang II) infusion,^8^, ^9^ thereby promoting proliferation of vascular smooth muscle cells, potentiating the development of hypertension, and extending endothelial dysfunction in conjunction with TNF‐α.^10^

TNF‐α, another proinflammatory cytokine primarily produced by T lymphocytes, shows heightened presence in various hypertension models and has been linked to increased production of reactive oxygen species (ROS) and vascular dysfunction.^11^, ^12^ The observed reduction in blood pressure upon TNF‐α blockade underscores the potential benefits of additional anti‐inflammatory therapies for individuals in the highest quartile of blood pressure, ultimately mitigating hypertension‐mediated organ damage.^13^

Conversely, interleukin‐10 (IL‐10) serves as an anti‐inflammatory cytokine, stimulating nitric oxide (NO) production and its exogenous administration reduce blood pressure in male spontaneously hypertensive rats (SHR).^14^ Notably, IL‐10 levels were found to be decreased in the vessels of Ang II‐induced hypertensive mice. As such, IL‐10 represents a potential therapeutic target for limiting the progression of Ang II‐induced aortic remodeling.^15^

Thus, acquiring a thorough comprehension of hypertension and its intricate connection with inflammation reveals promising new avenues for therapeutic interventions. Multiple studies have provided substantial evidence that monotherapy involving renin‐angiotensin system (RAS) inhibitors can modulate immune cell functions and regulate immune response mechanisms, not solely within the hypertensive patient population but across various other disease contexts as well.^16^, ^17^, ^18^ This compelling research suggests the potential of enhancing therapeutic outcomes and mitigating chronic inflammation through the synergistic use of RAS inhibitors in combination with antihypertensive medications from other pharmacological classes.^16^

Hence, the primary aim of this study was to explore the time‐dependent effects of a combined treatment regimen incorporating beta‐blockers and renin‐angiotensin system inhibitors on the dynamic variations of cytokines TNF‐α, IL‐6, and IL‐10. This research seeks to identify approaches with polytherapy to regulate the inflammatory processes within an experimental model of hypertension.

## 2 METHODS

Six‐months‐old male SHR rats (*N* = 144) with systolic blood pressure (SBP) of 171.8 ± 5.4 mm Hg, diastolic blood pressure (DBP) of 117.5 ± 6.7 mm Hg, and 6‐months‐old male WKY rats (*N* = 24) with an SBP of 112.8 ± 3.6 mm Hg and DBP of 83.3 ± 1.4 mm Hg were obtained from the animal facilities of the Institute of Physiology (UNAM). All experimental animals were provided with unrestricted access to both food and water and were accommodated within standard laboratory acrylic boxes, maintaining controlled environmental conditions. The experiment was designed according to the Official Norm NOM‐062‐ZOO‐1999, and this study was approved by the Institutional Committee for the Care and Use of Experimental Animals with registration code CICUAE‐FESC C17_15. Carcasses were handled in compliance with the Official Mexican Norm NOM‐087‐ECOL‐SSA1‐2002.

**FIGURE 1:**
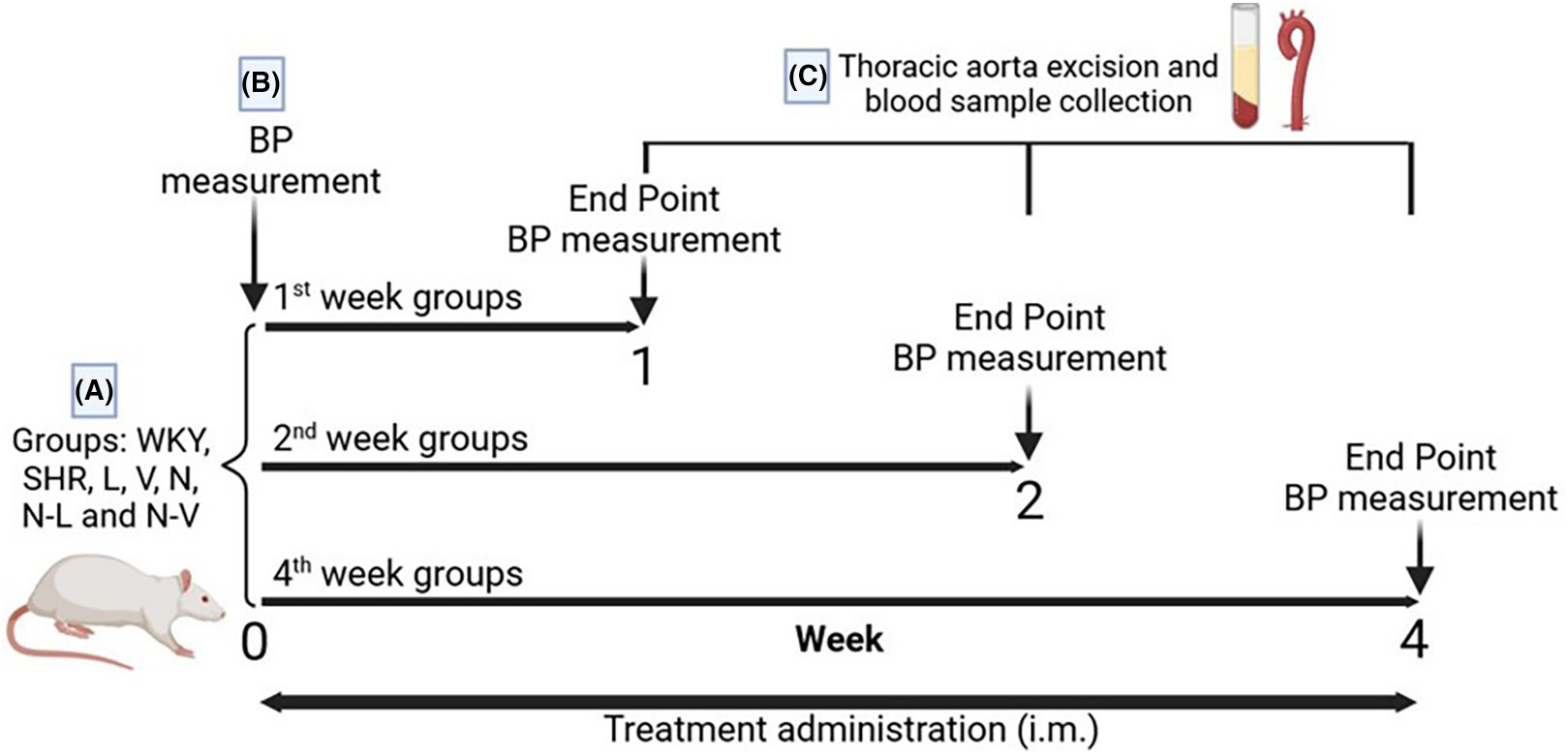
Experimental protocol. (A) Seven groups of treated rats were administered three different times: 1 week, 2 weeks, and 4 weeks. (B) In all groups, blood pressure was measured at the beginning and the end of the treatments. (C) At the end of each treatment time, the experimental animals were euthanized to obtain aorta and serum.

The experimental animals were randomly divided into seven groups (*n* = 6): (1) SHR + vehicle (saline solution); (2) SHR + nebivolol (0.8 mg/kg); (3) SHR + valsartan (1 mg/kg); (4) SHR + lisinopril (1 mg/kg); (5) SHR + nebivolol–valsartan (0.5–0.7 mg/kg), and (6) SHR + nebivolol–lisinopril (0.6–0.9 mg/kg) and (7) WKY + vehicle (saline solution). Lisinopril, nebivolol, and valsartan (Sigma‐Aldrich, MO, USA) were dissolved in saline solution and the concentration of each drug was adjusted to correspond to the dose administered in a volume of 0.1 mL. The treatments were administered intramuscularly once a day for 1, 2, and 4 weeks of treatment (Figure 1). Individual and combined doses were obtained from dose–response curves using SHR to achieve normotensive blood pressure values. These dosages have been employed in previous studies,^19^ demonstrating favorable outcomes in the effective management of blood pressure.

### 2.1 Blood pressure measurement

Systolic and diastolic pressures were determined by the tail‐cuff method during pharmacological treatment using SPAM and Sievart1 software (Instituto Nacional de Cardiología, Mexico). Before the study, the rats were trained using the tail‐cuff method for 1 week.

After treatment, blood samples (1 mL per rat) were collected from the tail vein for cytokine determination. The rats were euthanized in a CO_2_ chamber, and the aorta was quickly obtained and stored in RNAlater (Thermo Fisher Scientific, MA, USA) at −20°C for relative mRNA expression analysis.

### 2.2 mRNA identification by RT‐qPCR

Aortic RNA extraction was performed using TRIzol reagent method^15^ (Thermo Fisher Scientific, MA, USA). cDNA synthesis and polymerase chain reaction were performed using a QuantiNova SYBR® Green RT‐PCR Kit (QIAGEN, Gilden, Germany). The specific primers for the assay were as follows: TNF‐α sense: 3′‐CACCACGCTCTTCTGTCTACT‐5′, TNF‐α antisense: 3′‐AGATGATCTGAGTGTGAGGGTC‐5′; IL‐6 sense: 3′‐GAAATACAAAGAAATGATGGATGCT‐5′, IL‐6 antisense: 3′‐TTCAAGATGAGTTGGATGGTCT‐5′, IL‐10 sense: 3′‐CAATAACTGCACCCACTTCC‐5′ IL‐10 antisense: 3′‐ATTCTTCACCTGCTCCACTGC‐5′, and glyceraldehyde‐3‐phosphate dehydrogenase (GAPDH) was selected as the housekeeping gene; GAPDH sense: 3′‐AGACAGCCGCATCTTCTTGT‐5′, antisense: 3′‐CTTGCCGTGGGTAGAGTCAT‐5′. The primer BLAST tool was used for in silico validation of the primers for Rattus norvegicus (NIH, Bethesda, MD).

### 2.3 Cytokine determination

Blood samples were centrifuged at 3000 rpm for 15 min to obtain serum, which was frozen and kept at −80°C. TNF‐α, IL‐6, and IL‐10 plasma levels were assayed in triplicate using a Rat Tumor Necrosis Factor α ELISA Kit, Rat IL‐6 ELISA Kit, and Rat IL‐10 ELISA Kit (Sigma‐Aldrich, MO, USA), following the manufacturer's instructions.

### 2.4 Histological qualitative analysis

The aortic segments from each animal were fixed in 10% formalin, followed by paraffin embedding and precise sectioning at 5 μm thickness. Subsequently, deparaffinization and rehydration process was carried out to ensure optimal tissue preparation. The histological analysis was conducted using the classic hematoxylin and eosin stain, a method for assessing the overall histology of the aorta under a light microscope at 40× magnification. This approach provides only qualitative information for a comprehensive evaluation of the structural and cellular components within the aortic tissue.

### 2.5 Statistical analysis

Systolic and diastolic blood pressure values were expressed as mean ± 95% confidence intervals (95% CI), the mRNA results were expressed using the 2^−ΔΔCt^ method ±95% CI and cytokine levels are reported as mean values (pg/mL) ± 95% CI. Differences between SBP, DBP, relative mRNA expression, and cytokine serum levels were evaluated using one‐way ANOVA and Sidak's test. *p*‐values are derived from the comparison of the treatment groups versus the SHR group with vehicle. In all cases, statistical significance was set at *p* < .05. The graphs and statistical analyses presented in this study were generated using GraphPad Prism version 8.

### 2.6 Nomenclature of targets and ligands

Key protein targets and ligands in this article are hyperlinked to corresponding entries in http://www.guidetopharmacology.org, the common portal for data from the IUPHAR/BPS Guide to PHARMACOLOGY^20^ and are permanently archived in the Concise Guide to PHARMACOLOGY 2019/20.^21^

## 3 RESULTS

### 3.1 Blood pressure

**FIGURE 2:**
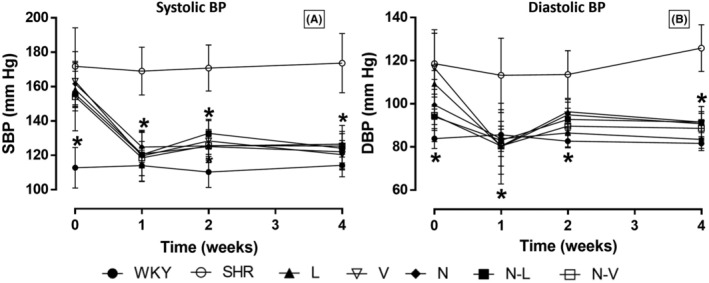
(A, B) Systolic and diastolic blood pressure values (mmHg) of rats treated for 1, 2, and 4 weeks. Data are expressed as mean [±95% confidence intervals]. *p < .001 versus SHR group. L, lisinopril; N, nebivolol; N–L, nebivolol–lisinopril; N–V, nebivolol–valsartan; SHR, SHR with vehicle; V, valsartan; WKY, Wistar Kyoto with vehicle.

Both polytherapy and monotherapy exhibited a substantial reduction in systolic blood pressure and diastolic blood pressure in SHR rats during the first week of treatment, as indicated by the significant results (Figure 2).

### 3.2 mRNA relative expression in aortic tissue

**FIGURE 3:**
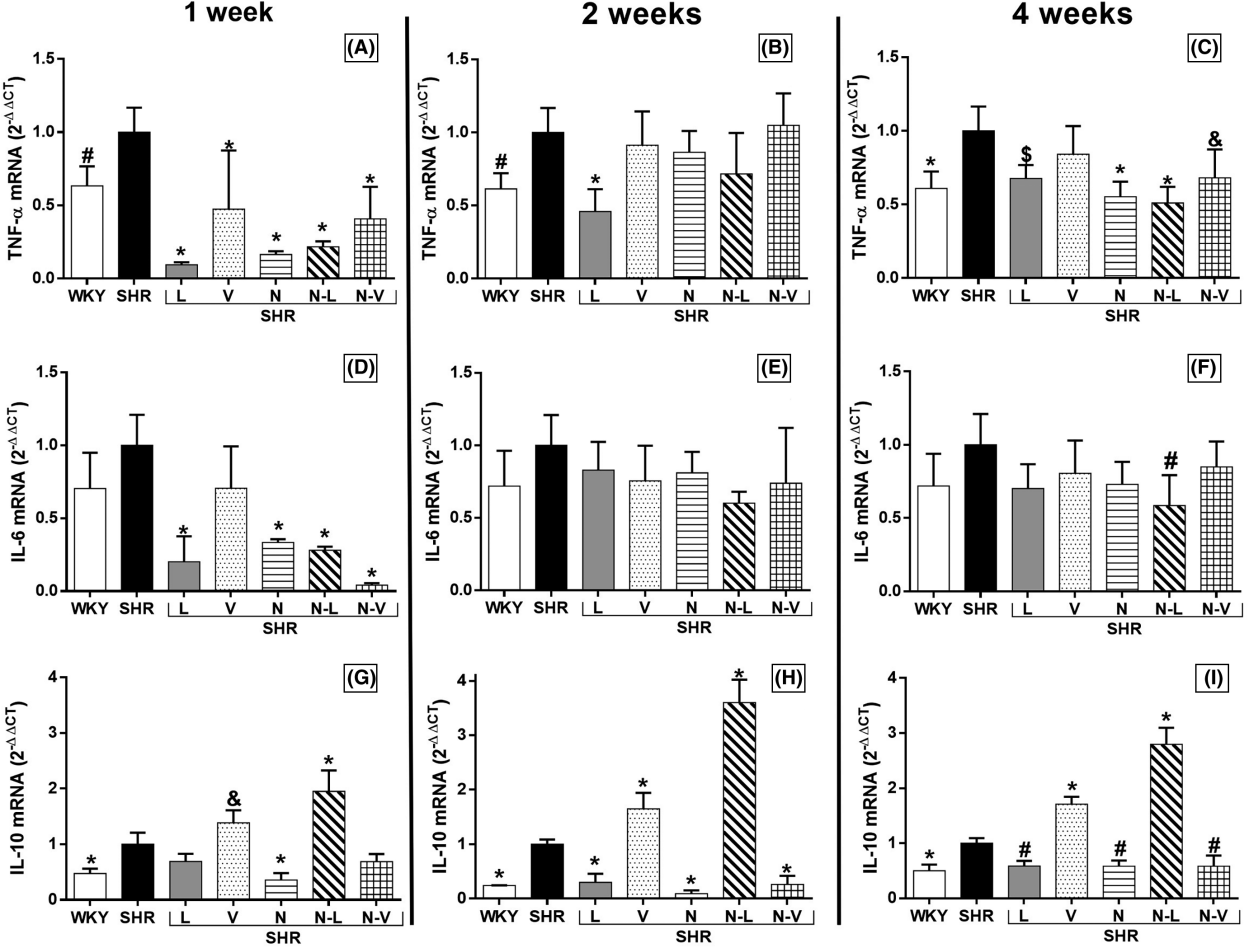
TNF‐α mRNA levels in aorta from rats treated for 1 week (A), 2 weeks (B), and 4 weeks (C). *p < 0.001, ^&^ *p* = .006, ^#^ *p* = .02, and ^$^ *p* = .005 versus SHR group. Interleukin‐6 mRNA levels in aorta from rats treated for 1 week (D), 2 weeks (E), and 4 weeks (F). *p < .001 and ^#^ *p* = .01 versus SHR group. Interleukin‐10 mRNA levels in aorta from rats treated for 1 week (G), 2 weeks (H), and 4 weeks (I). *p < .001, ^#^ *p* = .001, and ^&^ *p* = .03 versus SHR group. Results are reported as mean (±95% confidence interval). The treatments are lisinopril (L), valsartan (V), nebivolol (N), nebivolol–lisinopril (N–L), nebivolol–valsartan (N–V), Wistar Kyoto with vehicle (WKY), and SHR with vehicle (SHR).

All treatments led to a substantial reduction in the relative expression of TNF‐α during the first week, aligning the values with those observed in normotensive rats (Figure 3A). However, by the end of the second week, only the lisinopril treatment continued to maintain reduced TNF‐α mRNA levels (Figure 3B). By the fourth week, all treatments, with the exception of valsartan, sustained decreased TNF‐α expression (Figure 3C).

IL‐6 expression was significantly reduced in the first week with all treatments, except for valsartan (Figure 3D). In the second week (Panel E), there were no significant changes in IL‐6 expression observed with any of the treatments (Figure 3E). However, by the fourth week, only the nebivolol–lisinopril treatment led to a significant reduction in IL‐6 mRNA expression (Figure 3F).

There was an increase in IL‐10 expression with valsartan and nebivolol–lisinopril, and a decrease with nebivolol starting from the first week (Figure 3G). Additionally, there was a consistent decrease in IL‐10 in the second week (Figure 3H) and fourth week (Figure 3I) with lisinopril and nebivolol–valsartan.

### 3.3 Cytokine serum levels

**FIGURE 4:**
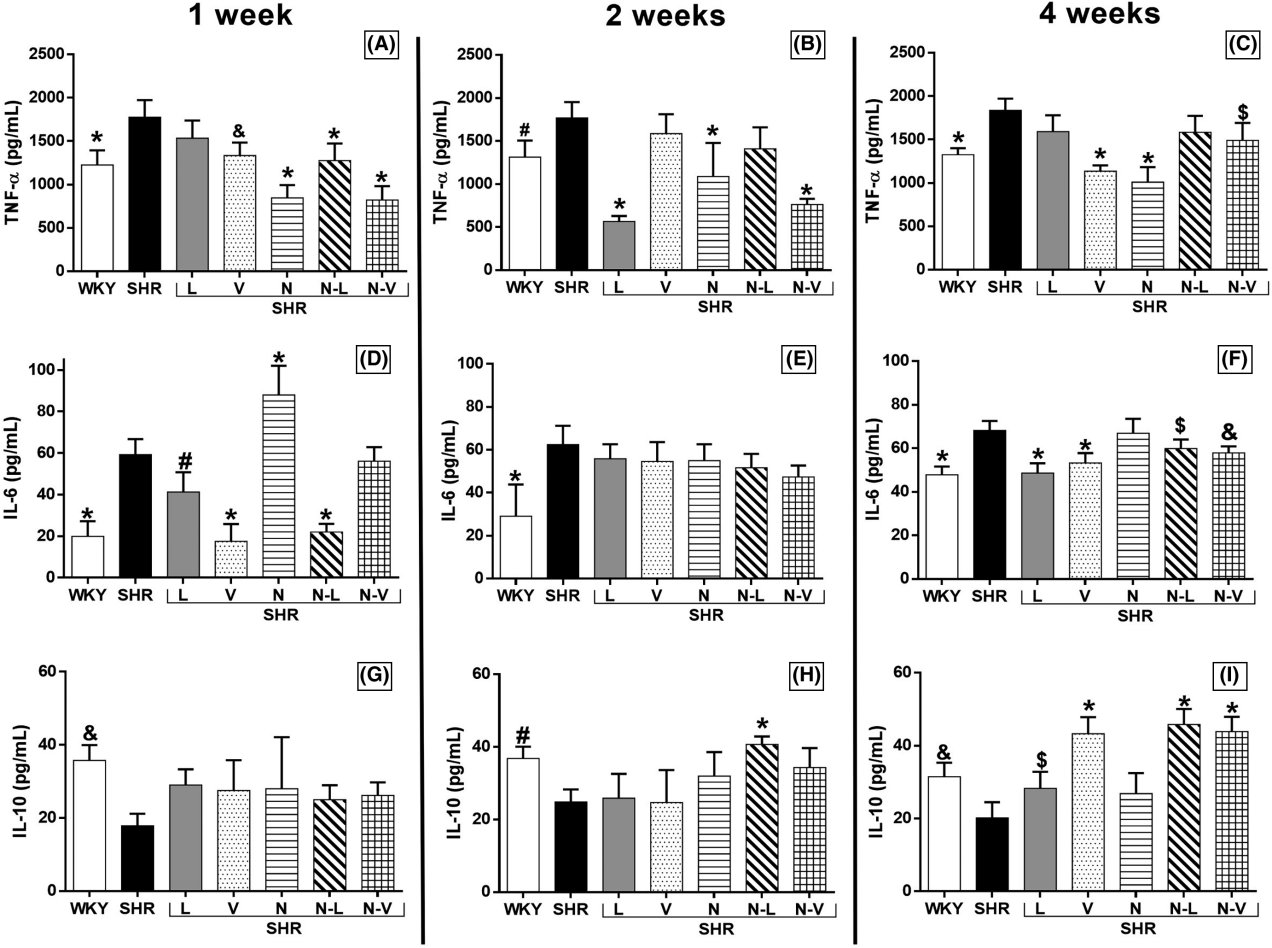
TNF‐α serum levels in rats treated for 1 week (A), 2 weeks (B), and 4 weeks (C). *p < .001, ^&^ *p* = .001, ^#^ *p* = .01, and ^$^ *p* = .006 versus SHR group. Interleukin‐6 serum levels in rats treated for 1 week (D), 2 weeks (E), and 4 weeks (F). *p < .001, ^#^ *p* = .01, ^$^ *p* = .04, and ^&^ *p* = .004 versus SHR group. Interleukin‐10 serum levels in rats treated for 1 week (G), 2 weeks (H), and 4 weeks (I). *p < .001, ^&^ *p* = .001, ^#^ *p* = .01, and ^$^ *p* = .04 versus SHR group. Results are reported as mean (±95% confidence interval). The treatments are lisinopril (L), valsartan (V), nebivolol (N), nebivolol–lisinopril (N–L), nebivolol–valsartan (N–V), Wistar Kyoto with vehicle (WKY), and SHR with vehicle (SHR).

All treatments led to a significant reduction in serum TNF‐α levels in SHR rats during the first week, except for lisinopril (Figure 4A). In the second week (Panel B), lisinopril, nebivolol, and nebivolol–valsartan demonstrated a reduction in serum TNF‐α levels (Figure 4B). By the fourth week, valsartan, nebivolol, and nebivolol–valsartan also significantly decreased serum TNF‐α levels (Figure 4C).

During the first week, lisinopril, valsartan, and nebivolol–lisinopril, except for nebivolol and nebivolol–valsartan, led to a reduction in serum IL‐6 levels compared to SHR rats (Figure 4D). In the second week, there were no significant changes in serum IL‐6 levels with lisinopril, valsartan, nebivolol, nebivolol–lisinopril, and nebivolol–valsartan (Figure 4E). However, by the fourth week, lisinopril, valsartan, nebivolol–lisinopril, and nebivolol–valsartan demonstrated significant reductions in serum IL‐6 levels (Figure 4F).

At first week of treatment, there were no significant changes in IL‐10 serum levels with lisinopril, valsartan, nebivolol, nebivolol–lisinopril, and nebivolol–valsartan (Figure 4G). In the second week, only nebivolol–lisinopril resulted in an increase in IL‐10 serum levels. Furthermore, in the fourth week, lisinopril, valsartan, nebivolol–lisinopril, and nebivolol–valsartan significantly increased serum IL‐10 levels, while nebivolol did not elicit a similar response (Figure 4H).

### 3.4 Aortic histological qualitative analysis

**FIGURE 5:**
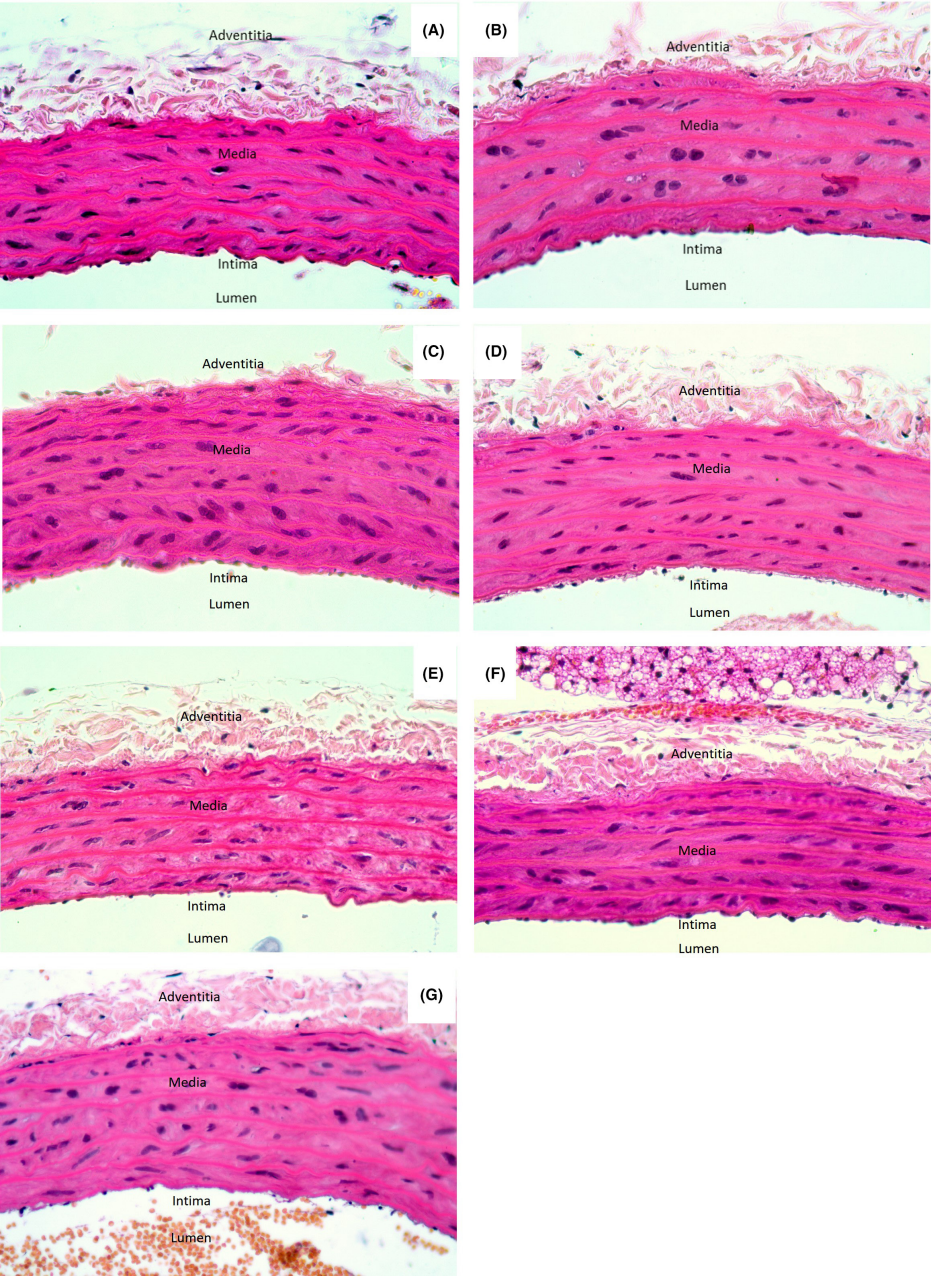
Histology of rat aortic tissue after 4 weeks of treatment and stained by hematoxylin and eosin at 40× magnification. Wistar Kyoto with vehicle (A), SHR with vehicle (B), lisinopril (C), valsartan (D), nebivolol (E), nebivolol–lisinopril (F) and nebivolol–valsartan (G).

Observation of the thoracic aortic tissues identified qualitative differences between the experimental groups, with the presence of two nuclei in several smooth muscle cells being evident in the SHR group (Figure 5B). Another feature identified was the apparent reduction of two nuclei in SHR rats treated with antihypertensives, mainly in the valsartan group (Figure 5D). Conversely, in the WKY group, no discernible alteration in the baseline cell division process was observed (Figure 5A).

## 4 DISCUSSION

The blood pressure reduction induced by treatments involving lisinopril, valsartan, nebivolol, and their combinations in SHR rats has been described in experimental models and individuals with hypertension.^22^, ^23^, ^24^, ^25^

Concerning proinflammatory cytokines, our study revealed elevated serum levels of TNF‐α and IL‐6, and increased TNF‐α mRNA expression was observed in SHR rats in comparison with the normotensive group (WKY), which may be associated with the increase in the expression and serum levels of TNF‐α and IL‐6 observed in hypertensive individuals compared to healthy counterparts.^26^ These elevations could be linked to peripheral endothelial dysfunction and an elevated risk of conditions such as myocardial infarction and heart failure.^26^, ^27^, ^28^ Additionally, both TNF‐α and IL‐6 have been identified as independent risk factors for elevated blood pressure in healthy patients.^29^ For this reason, it is imperative to modulate proinflammatory interleukin levels during the development of cardiovascular diseases such as hypertension. Regarding the reduction in IL‐10 serum levels in the SHR group in comparison to WKY, this is consistent with decreased plasma IL‐10 levels documented in patients with coronary artery disease and hypertension,^30^ as well as in various arterial hypertension models.^31^ The disparity in the effects of drug treatments on mRNA expression and serum interleukin concentrations implies an absence of consistent correlation between the two.^32^ Nevertheless, the quantification of both molecules should be deemed complementary, as they are both indispensable for comprehending cell function and elucidating drug mechanisms.^33^

The observed reduction in plasma IL‐6 levels under lisinopril treatment in the first and fourth weeks was in line with findings from another ACEi (captopril) treatment (80 mg/kg/day) in SHR rats over 12 weeks, where a reduction in IL‐6 plasma levels was associated with reduced mechanical stress on the vascular wall.^31^ Besides, captopril at 50 mg/day reduced plasma IL‐6 levels in hypertensive patients.^34^ These data may also explain why the nebivolol–lisinopril combination was able to reduce IL‐6 expression and serum levels at 4 weeks of treatment, which was not observed with nebivolol monotherapy, and thus the effect would be due to ACEi. On the other hand, the increase in serum levels induced by lisinopril could be related to the effects described with the administration of ACEis, such as captopril and enalapril in rodent models.^35^, ^36^ Interestingly, the nebivolol–lisinopril combination proved to be even more effective in increasing not only serum IL‐10 levels but also the expression of IL‐10 starting from the second week of treatment. This enhanced effect could be attributed to the synergistic actions of ACE inhibitors^16^ and nebivolol on this anti‐inflammatory cytokine.

The decrease in serum TNF‐α and IL‐6 produced by valsartan at 1 and 4 weeks of treatment may be associated with the effects previously described in patients with essential hypertension, who showed a reduction in blood pressure and serum TNF‐a and IL‐6 cytokines after treatment with valsartan (80 mg/day) for 3 months.^37^ Other angiotensin receptor antagonists, such as olmesartan, have similarly decreased TNF‐α and IL‐6 levels in hypertensive patients.^38^, ^39^ Additionally, telmisartan reduced TNF‐α‐induced IL‐6 mRNA expression in T lymphocytes and vascular smooth muscle cells (VSMC).^40^, ^41^, ^42^ These findings highlight the potential anti‐inflammatory effects of angiotensin receptor antagonists (ATR) and their promising role in combination therapy. Furthermore, the increase in IL‐10 mRNA expression throughout the course of treatment, this elevation in IL‐10 has also been observed in a murine model of atherosclerosis that received valsartan (10 mg/kg) for 8 weeks^43^ and in a rat colitis model with valsartan (160 mg/L) treatment for 1 month.^44^ Conversely, when patients with renal disease and hypertension were treated with valsartan at a daily dose of 100 mg for 1 week, there were no significant changes in IL‐10 levels.^45^ These observations imply that the effects of valsartan on this cytokine are related to the duration of treatment.

It has been reported that nebivolol at doses of 5 and 10 mg/kg for 3 weeks does not modify IL‐6 or TNF‐α lipopolysaccharide (LPS)‐induced expression in SHR rats.^46^ Contrary to this, in our results nebivolol reduced serum TNF‐α levels, this difference in results could be associated with the use of LPS as a stimulator of the inflammatory process in their study, in contrast to the use of a hypertension model with a disease‐derived inflammatory process. Moreover, it has been demonstrated that nebivolol has anti‐inflammatory effects, decreases arterial stiffness, and improves endothelial function.^47^ For this reason, it is important to propose the use of antihypertensive combinations with the use of nebivolol. In this regard, other β‐adrenergic blockers, such as carvedilol, have been reported to reduce the levels of inflammatory markers in patients with heart failure, which has been related to the antioxidant effects of the drugs.^48^ While propranolol significantly suppressed IL‐6 expression in vascular endothelial cell culture.^49^ Based on our findings, nebivolol did not induce any significant alterations in serum IL‐10 levels, this outcome appears to parallel the results observed in serum samples from obese rats who received nebivolol (5–10 mg/kg) for 8 weeks.^50^ In contrast, when mice were treated with the same doses of nebivolol for a duration of 2 weeks, there was a noticeable increase in IL‐10 levels specifically within brain tissue.^51^ This intriguing disparity in the effects of this beta‐blocker suggests that its impact may be influenced by the type of tissue involved and the specific pathology under consideration.

In the case of nebivolol–valsartan, the reduction in TNF‐α mRNA expression and serum TNF‐α levels was observed at the first and fourth weeks, this could be attributed to the effect of valsartan, since drugs targeting the renin‐angiotensin system can directly suppress inflammation by inhibiting proinflammatory cytokine synthesis in human coronary artery endothelial cells.^49^ Furthermore, a combination of valsartan (80 mg/day) with ACEi (18 mg/day) for 6 months has been shown to protect vascular endothelial function and inhibit NF‐κB overactivity in endothelial cells by reducing serum TNF‐α and IL‐6 levels.^52^ In addition, ATR antagonists like valsartan have decreased the levels of inflammatory markers in healthy individuals, as well as those with hypertension and heart failure.^53^, ^54^, ^55^ Additionally, the rise in serum IL‐10 levels resulting from the combined administration of nebivolol and valsartan may be attributed to the impact of valsartan on IL‐10, an effect consistent with our study findings and reported in other models.^43^ Notably, such an effect was not evident when nebivolol was used as a standalone therapy. This suggests that the combination of valsartan with nebivolol may benefit from the anti‐inflammatory effects of RAS inhibitors with the antioxidant properties of nebivolol.

Concerning the alterations noted in the aortic wall, the prevalence of polyploidy in aortic tissue reaches up to 7% in healthy adults.^56^ However, a notable increase in the number of nuclei of aortic VSMCs was discerned in the SHR group when compared to the WKY group. This increase has been linked to cell hypertrophy induced by the physiological stress of hypertension,^57^, ^58^ which could be related to the capacity of angiotensin II to expedite the cell cycle in SHR, consequently leading to changes in VSMC.^59^ Conversely, there was an apparent reduction in the number of nuclei observed in the aortic smooth muscle cells following valsartan treatment and its combination with nebivolol, which could be related to the blockade of the AT_1_ receptor and potentially decelerate the impact of angiotensin II.^60^ Hence, it is important to investigate the cellular division processes linked to antihypertensive treatments within the vascular tissues using specific techniques.

The potential mechanism underlying the reduction in proinflammatory cytokines TNF‐α and IL‐6 when treated with RAS inhibitors is associated with their respective actions: lisinopril reduces the production of Ang II, whereas valsartan blocks Ang II receptors. This could be explained by the fact that Ang II stimulates the production of TNF‐α and IL‐6 via the AT1 receptor in monocytes, macrophages, and VSMC.^10^, ^61^, ^62^ Consequently, the blockade of angiotensin II would indeed exert an influence on the production of these cytokines. In addition, Ang II has been implicated in triggering an inflammatory response in cardiac tissue via the activation of the nuclear factor NF‐κB.^63^, ^64^ Moreover, Ang II has been linked to heightened subclinical inflammation in both experimental models and patients with coronary heart failure.^65^ Hence, RAS inhibitors should be regarded as an integral component of antihypertensive treatment aimed at effectively managing inflammation. Furthermore, the combination of these RAS inhibitors with nebivolol would not only slow down the proinflammatory process^50^, ^66^ but would also have an anti‐inflammatory and antioxidant effect characteristic of this beta‐blocker, which regulates the production of ROS by NADPH oxidase.^67^

In conclusion, the use of combinations of RAS inhibitors with the latest generation of beta‐blockers offers adequate blood pressure control together with a time‐dependent modulating effect on proinflammatory cytokines such as TNF‐α and IL‐6 during the treatment period and may also promote some modulatory mechanisms of the inflammatory process through IL‐10 during the treatment.

## AUTHOR CONTRIBUTIONS

Lezama‐Martinez D, Hernandez‐Campos ME contributed to the design, implementation of the research, and supervised the project. Garrido‐Farina German‐Isauro, Fonseca‐Coronado S, Ramirez‐Hernandez D, and Flores‐Monroy J to the analysis of the results and to the writing of the manuscript.

## FUNDING INFORMATION

This work was supported by grants from UNAM‐PAPIIT IN217122, IN202022, FESC CI‐2259, and CI‐2211.

## CONFLICT OF INTEREST STATEMENT

The authors report no conflicts of interest.

## ETHICS STATEMENT

This study adhered to the guidelines outlined in the NIH Guide for the Care and Use of Laboratory Animals, as well as the Mexican Official Norm NOM‐062‐ZOO‐1999. Approval for the study was granted by the Institutional Committee for the Care and Use of Experimental Animals under registration code CICUAE‐FESC C17_15. Carcasses were handled in compliance with the Official Mexican Norm NOM‐087‐ECOL‐SSA1‐2002.

## Data Availability

The data that support the findings of this study are available on request from the corresponding author.
